# The Effect of Different Vegetable Oils on Cedar Wood Surface Energy: Theoretical and Experimental Fungal Adhesion

**DOI:** 10.1155/2022/9923079

**Published:** 2022-01-13

**Authors:** Fadoua Bennouna, Moulay Sadiki, Soumya Elabed, Saad Ibnsouda Koraichi, Mohammed Lachkar

**Affiliations:** ^1^Laboratory of Microbial Biotechnology and Bioactive Molecules, University Sidi Mohammed Ben Abdellah, Faculty of Science and Technology, Po. Box 2202, Fez 30007, Morocco; ^2^Engineering Laboratory of Organometallic, Molecular Materials and Environment, University Sidi Mohammed Ben Abdellah, Faculty of Science, Po. Box 1796, Fez 30000, Morocco; ^3^Laboratory of Molecular Engineering, Valorization and Environment, University Ibn Zohr, Faculty Polydisciplinary of Taroudant, BP. 271, Taroudant 83000, Morocco; ^4^Regional University Centre of Interface, University Sidi Mohammed Ben Abdellah, Po. Box 2626, Fez 30000, Morocco

## Abstract

Despite having been used for ages to preserve wood against several effects (biological attack and moisture effects) that cause its degradation, the effect of vegetable oils on the cedar wood physicochemical properties is poorly known. Thus, in this study, the hydrophobicity, electron-acceptor (*γ*^+^), and electron-donor (*γ*^−^) properties of cedar wood before and after treatment with vegetable oils have been determined using contact angle measurement. The cedar wood has kept its hydrophobic character after treatment with the different vegetable oils. It has become more hydrophobic quantitatively with values of surface energy ranged from −25.84 to −43.45 mJ/m^2^ and more electron donors compared to the untreated sample. Moreover, the adhesion of four fungal strains (*Penicillium commune* (PDLd”), *Thielavia hyalocarpa*, *Penicillium commune* (PDLd10), and *Aspergillus niger*) on untreated and treated cedar wood was examined theoretically and experimentally. For untreated wood, the experimental adhesion showed a positive relationship with the results obtained by the extended Derjaguin–Landau–Verwey–Overbeek (XDLVO) approach which found that all fungal strains could adhere strongly to the cedar wood material. In contrast, this relationship was not always positive after treatment. The Environmental Scanning Electron Microscopy (ESEM) has shown that *P. commune* (PDLd10) and *A. niger* were found unable to adhere to the wood surface after treatment with sunflower and rapeseed oils. In addition, the results showed that the four fungal strains' adhesion was decreased with olive and linseed oils treatment except that of P. *commune* (PDLd10) treated with linseed oil.

## 1. Introduction

Cedar wood is one of the oldest building materials in Morocco. Its use dates back to the Idrisids dynasty for building historical monuments of the Medina of Fez (mosques, schools, houses, etc.). In addition to its numerous qualities (strength, visual appearance, and the good thermal insulation properties), wood hygroscopicity is an important property considered as a negative characteristic. Indeed, wet conditions create a very favorable environment for the growth of various wood-degrading biological organisms (fungi, bacteria, and insects) [1]. These latter adhere to this material and form biofilms that cause a discoloration on building materials [2–4], reduction of wood durability due to structural and chemical changes [5], degradation of the historical monuments, and therefore, a loss of our cultural heritage. The most important step in the biofilm formation process is the microbial adhesion to the substrate surface. It involves mainly the Van der Waals, electrostatic, and acid-base interactions. These latter depend on the physicochemical characteristics of material and the microbial surface, especially hydrophobicity, surface tension, and electron-donor-electron-acceptor properties [6–8]. Therefore, it is very important to understand the physicochemical characteristics of microbial cell and solid surfaces in order to predict the microbial adhesion.

The prediction of microbial adhesion on the surface of different materials was studied using the extended DLVO (XDLVO) theory which introduces other non-DLVO forces as polar forces of Lewis (acid-base interactions) compared to the classical DLVO theory which considers only the van der Waals and electrostatic forces [9, 10]. In addition, it was claimed that the XDLVO approach may be the promising model to explain the experimental findings of microbial adhesion obtained by the environmental scanning electronic microscopy (ESEM) technique.

Previous works have explored the effect of the plasma polymerization technique [11], thermal treatment [12–14], and plant extracts [15] on the wood surface physicochemical characteristics. In addition, some studies have been conducted on the chemistry of vegetable oils within wood [16]. Vegetable oils are widely used as raw renewable resources in biopolymer synthesis, due to their low toxicity and ease of multiple functionalization [17]. Hence, their versatile compositions permit them to engage in various types of chemical reactions for generating new monomers, with epoxidation being one of the main and widely applied chemical transformations [18, 19]. The use of epoxy functional vegetable oil and reactive UV-absorber as pretreatment for Scots pine and sapwood [20] and on the effect of linseed and tung oils on wood and water uptake has been reported [21]. However, there are no investigations on the effect of vegetable oils on the cedar wood physicochemical properties and their antiadhesive activity against fungi associated with its deterioration. Vegetable oils are very complex structures, widely applied in coatings, which undergo as many as 9 modifications reaction types (epoxidation, transesterification, glycerolysis, amidation, etc.). Thus, the purpose of this work is

- To study the effect of sunflower oil, rapeseed oil, linseed oil, olive oil, and argan oil on the cedar wood physicochemical properties. These vegetable oils are ecofriendly and low priced, enhance the color of wood, and offer it excellent protection.
- To predict fungal spores adhesion on treated cedar wood using theoretical and experimental evaluation.

## 2. Materials and Methods

### 2.1. Vegetable Oils

Vegetable oils are considered as being a natural resource. In addition to the fact that they do not have toxic chemical effects on the environment, vegetable oils are nonvolatile and low priced [22]. The oils used in this study were sunflower oil, rapeseed oil, linseed oil, olive oil, and argan oil, all of which were commercially available.  Table 1 provides an overview of the chemical composition of each vegetable oil.

### 2.2. Fungal Strain Growth Conditions

Four fungi (*Penicillium commune* (PDLd”)*, Penicillium commune* (PDLd10)*, Thielavia hyalocarpa*, and *Aspergillus niger*) were used in this study. They were isolated from cedar wood decayed from an old house in the old Medina of Fez (Morocco) and identified in the laboratory of microbial biotechnology [25, 26]. These strains were grown in a malt-extract-agar medium at 25°C for 10 days. The fungal spores were then collected using sterile solution of KNO_3_ (0.1 M). The spore suspensions were centrifuged at 7000 rpm for 15 min at 4°C. The pellets obtained were washed with sterile KNO_3_ and resuspended in the same solution to a final concentration of 10^7^-10^8^ spores/mL.

### 2.3. Wood Preparation

The cedar wood samples (*Cedrus atlantica*) were provided from a woodworking shop in Fez city, Morocco, September 2015. The roughness of the wood samples (30 × 10 × 4 mm) was set in a range from 0.8 to 1 *μ*m using a rugosimeter. At the end, the samples were cleaned with distilled water, oven-dried, and then, autoclaved at 121°C for 20 min.

### 2.4. Wood Treatment

20 *μ*L of pure vegetable oil was deposited to the cedar wood surface at room temperature (25 ± 2°C) for 1 h so that the surface of wood is dried [15]. The samples were analyzed with contact angle measurements in order to evaluate the effect of each vegetable oil on the cedar wood physicochemical properties. Experiments were conducted in duplicate.

### 2.5. Contact Angle Measurements and Surface Tension Components of Fungal Strains and Wood Surfaces

The Lifshitz–nan der Waals, acid-base, and surface free energy of fungal strains and wood samples were calculated from contact angle measurements which were realized by the sessile drop method using a goniometer (GBX Instruments) [27]. Three measurements of contact angles were made on each samples using three liquids (of which two must be polar: water (W) and formamide (F) and one nonpolar: diiodomethane (D)) with well-known surface energy components (Table 2) [29].

For *A. niger* strain, the contact angle measurements were performed as described by Busscher et al. [30]. For that, 10 mL of the spore suspension already prepared was filtered on a cellulose acetate membrane filter of 0.45 *μ*m. After a good drying of the filters (air drying for 30 min at room temperature), contact angles were measured. The measurements of the contact angle were carried out in duplicate for two different fungal lawns. The contact angle measurements of *T. hyalocarpa* and *P. commune* (PDLd10 and PDLd”) were determined by [31, 32] following the same methodology mentioned above.

Once the contact angles were measured, the Lifshitz–van der Waals and acid-base surface tension components were obtained by the three equations of the following form [8]:

(1) $$\begin{array}{c} \gamma_{L}\left(\cos\theta +1\right)=2{\left(\gamma_{S}^{LW}\gamma_{L}^{LW}\right)}^{1/2}+2{\left(\gamma_{S}^{+}\gamma_{L}^{-}\right)}^{1/2}+2{\left(\gamma_{S}^{+}\gamma_{L}^{-}\right)}^{1/2}, \end{array}$$

where *θ*: the contact angle, *γ*^*LW*^: the van der Waals free energy component, *γ*^+^: the electron-acceptor component, *γ*^−^: the electron-donor component, and *S* and *L* stand for the solid surface and liquid phases, respectively.

The surface free energy is formulated as

(2) $$\begin{array}{c} \gamma_{S}^{\text{Tot}}=\gamma_{S}^{LW}+\gamma_{S}^{AB}, \end{array}$$

where  *γ*_*S*_^*AB*^=2(*γ*_*S*_^−^*γ*_*S*_^+^)^1/2^ is the Lewis acid-base component.

The fungal strains and wood samples' hydrophobicity was evaluated through contact angle measurements and by the approach in [29]. In this approach, the degree of hydrophobicity of a specific material can be defined as the free energy of interaction between two entities of this latter when immersed in water (w): ΔGiwi. So, we said that the material is hydrophilic whether the interaction between the two entities is lower than the interaction of each entity with water (ΔGiwi > 0); otherwise, the material is considered as hydrophobic ∆Giwi < 0. ΔGiwi is calculated as reported in the following formula:

(3) $$\begin{array}{c} \Delta Giwi=-2\gamma_{iw}=-2\left[{\left({\left(\gamma_{i}^{LW}\right)}^{1/2}-{\left(\gamma_{w}^{LW}\right)}^{1/2}\right)}^{2}+2\left({\left(\gamma_{i}^{+}\gamma_{i}^{-}\right)}^{1/2}+{\left(\gamma_{w}^{+}\gamma_{w}^{-}\right)}^{1/2}-{\left(\gamma_{i}^{+}\gamma_{w}^{-}\right)}^{1/2}-{\left(\gamma_{w}^{+}\gamma_{i}^{-}\right)}^{1/2}\right)\right]. \end{array}$$

### 2.6. Total Free Energy of Interaction: The Extended DLVO Theory

The classical DLVO theory considers only the forces of van der Waals and electrostatic. This theory was expanded in [10] to take into account polar interaction called as non-DLVO force. The total free energy is the sum of the interfacial energies of Lifshitz–van der Waals Δ*G*^*LW*^ and Lewis Δ*G*^*AB*^. In this work, the electrical interactions were ignored because of the higher ionic strength the suspending solution KNO_3_ used (0.1 M) [33, 34].

(4) $$\begin{array}{c} \Delta G^{\text{Total}}=\Delta G^{LW}+\Delta G^{AB}. \end{array}$$

The interaction between two flat surfaces *i* and *j* (microbial cell and substratum), separated by a medium (water), is written as

(5) $$\begin{array}{c} \Delta G^{LW}=-2\;\left(\sqrt{\gamma_{i}^{LW}}-\sqrt{\gamma_{w}^{LW}}\right)\left(\sqrt{\gamma_{j}^{LW}}-\sqrt{\gamma_{w}^{LW}}\right),\\ \Delta G^{AB}=-2\;\left[\sqrt{\gamma_{w}^{+}}\left(\sqrt{\gamma_{i}^{-}}+\sqrt{\gamma_{j}^{-}}-\sqrt{\gamma_{w}^{-}}\right)+\sqrt{\gamma_{w}^{-}}\left(\sqrt{\gamma_{i}^{+}}+\sqrt{\gamma_{j}^{+}}-\sqrt{\gamma_{w}^{+}}\right)-\sqrt{\gamma_{i}^{+}\gamma_{j}^{-}}-\sqrt{\gamma_{i}^{-}\gamma_{j}^{+}}\right]. \end{array}$$

Fungal spore attachment is favored if Δ*G*^Total^ is negative and unfavored in the opposite case (Δ*G*^Total^ is positive).

### 2.7. Adhesion Experiments

After treating the wood samples with vegetable oils tested, they were immersed in spore suspension at a concentration of 10^7^ spores/mL for 10 h at 25°C [26]. At the end of the contract period, the samples were rinsed three times with sterile distilled water to remove spores that have not adhered to the wood surface.

### 2.8. Environmental Scanning Electron Microscopy Analysis

All wood samples were analyzed by Environmental Scanning Electron Microscopy (ESEM) Quanta 200 equipped with a tungsten filament. The ESEM images obtained present precious information about the antiadhesion effect of each vegetable oil tested as well as the adhesion of strains studied. The percentage of fungal spores adhered to the wood surface was determined by the MATLAB software program® [33].

## 3. Results and Discussion

### 3.1. Effect of Vegetable Oils on the Physicochemical Properties of Cedar Wood

Table 3 summarizes the contact angles values and the surface energies, together with their *γ*^*LW*^, *γ*^*AB*^, *γ*^−^, *γ*^+^, and *γ*^Tot^ of untreated and treated cedar wood. In line with the work in [35] and the approach in [29, 36], the untreated cedar wood surface was hydrophobic qualitatively with values of *θ*_*W*_ = 87.13 ± 0.15° and quantitatively with values of ∆Giwi = −59.29 mJ/m^2^. Also, the results showed that the degree of hydrophobicity has not changed much qualitatively and quantitatively even after treatment of the wood surface with sunflower, rapeseed, linseed, olive, and argan oils. Indeed, the cedar wood has kept its hydrophobic character after treatment with values of the water contact angles ranged from *θ*_*W*_ = 64.95 ± 0.24 to 73.95 ± 0.29° and values of surface energy ranged from −25.84 to −43.45 mJ/m^2^. These findings confirm those found by Jiang and Kamdem [37] who reported that the northern red oak wood has kept its hydrophobic character after treatment with a copper ethanolamine solution (*θ*_*W*_ ˃ 100°). However, unlike our results, several studies have shown that the untreated cedar wood sample, which was hydrophobic, has become more hydrophilic after treatment with essential oil components [38, 39] and *Thymus vulgaris* extracts [15, 31].

The results showed also the increasing of electron-donor character after treatment with values ranging from 10.66 to 17.66 mJ/m^2^ compared to the initial value of the untreated wood which is 4.47 mJ/m^2^. The values of the electron-acceptor character are almost negligible. These statements are consistent with those found by Mohammed-Ziegler et al. [40], who noted that the electron-donor character of European oak wood treated with octadecyltrichlorosilane and chlorotrimethylsilane was higher (*γ*^−^ = 2.3 mJ/m^2^ and *γ*^−^ = 5.4 mJ/m^2^, respectively) than that of the control (*γ*^−^ = 0.3 mJ/m^2^).

### 3.2. Physicochemical Properties of Fungal Cells

The physicochemical properties (hydrophobicity, the surface free energy, acid-base, the Lifshitz–van der Waals component, and electron-donor and electron-acceptor parameters) of the four fungal strains studied are represented in Table 4. *Penicillium commune* (PDLd”), *Thielavia hyalocarpa*, and *Penicillium commune* (PDLd10) are qualitatively and quantitatively hydrophilic. The water contact angle values ranged from 36.11 to 41.90° and 8.28 to 37.12 mJ/m^2^ for the surface free energy. *Aspergillus niger* is qualitatively hydrophilic and quantitatively hydrophobic (∆Giwi = −12.57 mJ/m^2^). Interestingly, it can also be seen from Table 4 that all strains have high values of electron-donor character (*γ*^−^) and low values of electron-acceptor character (*γ*^+^). Several studies have shown that almost all microbial cells have electron-donor character, especially those presented in [41].

The microbial cell surface properties depend mainly on its chemical composition, especially the protein/carbohydrate ratio [42–44]. In fact, spores which have greater protein/carbohydrate ratio have a rough surface and are more hydrophobic, unlike those with lower protein/carbohydrate ratio [45].

### 3.3. Theoretical Evaluation of the Adhesion of Four Fungal Strains to the Cedar Wood Surface before and after Treatment

A good understanding of the microbial adhesion phenomenon cannot be carried out without taking into account the mechanisms involved in the interaction between the microbial cell and substrate. As previously mentioned, the aim of this theoretical evaluation was to provide information about the adhesion of *P. commune* (PDLd”), *T. hyalocarpa*, *P. commune* (PDLd10), and *A. niger* to the cedar wood surface before and after treatment with different vegetable oils and, thus, determine all the forces that govern this adhesion.

The results obtained in Table 5 show that all fungal strains could adhere to the untreated wood surface (∆*G*^Tot^ ˂ 0). *A. niger* is the strain that adheres more to the wood surface with a value of ∆*G*^Tot^ = −20.06 mJ/m^2^, and *P. commune* (PDLd10) is the least-adhering strain with a value of ∆*G*^Tot^ = −3.49 mJ/m^2^. Moreover, the values of the ∆*G*^*AB*^ component of the four fungal strains were higher than those of the ∆*G*^*LW*^ component. Indeed, for *P. commune* (PDLd10) and *Aspergillus niger*, the theoretical adhesion should be governed only by short-range forces (the acid-base interactions) because ∆*G*^*AB*^ is negative and ∆*G*^*LW*^ is positive. In contrast, for *P. commune* (PDLd”) and *T. hyalocarpa*, the theoretical adhesion should be governed more by short-range forces than long-range forces (both have a negative value, but ∆*G*^*AB*^ > ∆*G*^*LW*^).

In addition, we noted that, after treatment with sunflower, rapeseed, linseed, and olive oils, the adhesion was not favorable in most cases. In other cases, the adhesion was still favorable, but it was decreased. Indeed, the adhesion was not favorable after treatment with olive oil for both *P. commune* (PDLd”) and *P. commune* (PDLd10) with values of ∆*G*^Tot^ = 1.61 mJ/m^2^ and ∆*G*^Tot^ = 4.27 mJ/m^2^, respectively, and it was decreased after treatment with sunflower oil for *A. niger* (∆*G*^Tot^ = −1.26 mJ/m^2^) and rapeseed oil for *Thielavia hyalocarpa* (∆*G*^Tot^ = −4.55 mJ/m^2^). Moreover, the results showed that, for all strains studied, after treatment with argan oil, the adhesion is much more favorable than before treatment and the theoretical adhesion is governed by ∆*G*^*AB*^. By contrast, the theoretical adhesion for the strains studied is governed by ∆*G*^*LW*^ after treatment with sunflower, rapeseed oil, linseed, and olive oils.

### 3.4. Experimental Adhesion of Fungal Strains to the Cedar Wood Surface before and after Treatment

**Figure 1:**
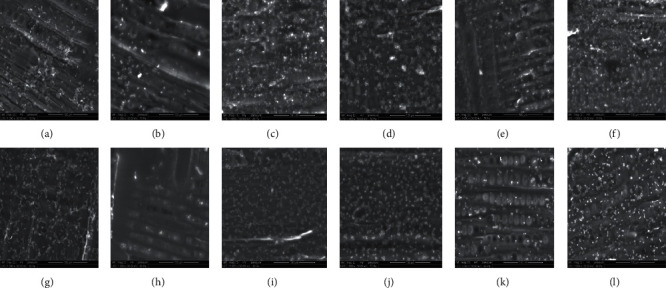
Electron micrographs of *Penicillium commune* (PDLd”) (a–f) and *Thielavia hyalocarpa* (g–l) spores adhered on to untreated and treated wood, visualized by environmental scanning electron microscopy. (a, g) untreated wood, (b, h) olive oil treatment, (c, i) sunflower oil treatment, (d, j) rapeseed oil treatment, (e, k) linseed oil treatment, and (f, l) argan oil treatment.

**Figure 2:**
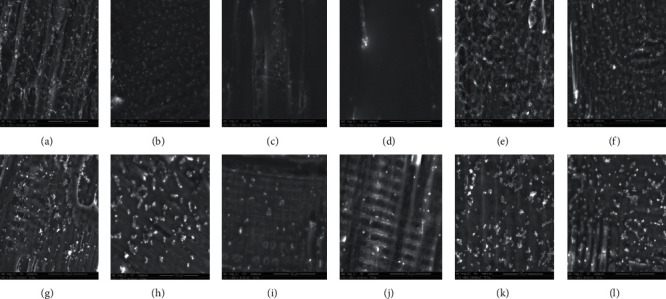
Electron micrographs of *Penicillium commune* (PDLd10) (a–f) and *Aspergillus niger* (g–l) spores adhered on to untreated and treated wood, visualized by environmental scanning electron microscopy. (a, g) untreated wood, (b, h) olive oil treatment, (c, i) sunflower oil treatment, (d, j) rapeseed oil treatment, (e, k) linseed oil treatment, and (f, l) argan oil treatment.

As shown in Figures 1(a) and 1(g) and 2(a) and 2(g), *P. commune* (PDLd”), *T. hyalocarpa*, *P. commune* (PDLd10), and *A*. *niger* are able to adhere greatly to the cedar wood with a percentage of adhesion of 26.78%, 13.95%, 22.48%, and 22.32%, respectively. They were found dispersed as single, pairs, and into clusters of spores. These statements corroborate with those found by Sadiki et al. and El Abed et al. [31, 46] who studied the adhesion of fungal spores associated with the deterioration of cedar wood on the same wood species.

All fungal strains studied, which have a hydrophilic character, have adhered to the untreated cedar wood surface that has a hydrophobic character. These results are not corroborated with the correlation that said hydrophobic cells adhere more to hydrophobic surfaces and hydrophilic ones, as well as the results found in [7] showed that the hydrophobicity of several microorganisms is correlated with the adhesion to the hydrophobic solid surface. However, others authors reported that the acid-base interactions play a very important role in the microbial adhesion on a support beside the hydrophobicity character [47–49].

Interestingly, it can be seen from Figures 1(b) and 1(h) and 2(b) and 2(h) that the adhesion of all fungal strains was influenced by olive oil treatment, especially for *P. commune* (PDLd”) and *T. hyalocarpa* with the percentage of adhesion of 12.54% and 10.31%, respectively. Olive oil treatment has decreased the percentage of adherence from 22.48 to 10.91% for *P. commune* (PDLd10) and from 22.32 to 18.91% for *A. niger*. After treatment with sunflower oil, *P. commune* (PDLd10) and *A. niger* were found unable to adhere to the wood and presented low percentages of adhesion (8.10% and 9.09%, respectively) (Figures 2(c) and 2(i)). The same was detected after treatment with rapeseed oil with 6.05% and 11.31% of spores adhered (Figures 2(d) and 2(j)). However, the adhesion of *P. commune* (PDLd”) and *T. hyalocarpa* has increased after treatment with sunflower (28.49% for *P. commune* (PDLd”) and 20.96% for *T. hyalocarpa*) and rapeseed oil (36.86% for *P. commune* (PDLd”) and 31.73% for *T. hyalocarpa*). Linseed oil treatment has decreased the percentage of adherence for *P. commune* (PDLd”), *T. hyalocarpa*, and *A. niger* with percentages of adhesion of 13.72%, 5.72%, and 12.56%, respectively, and increased from 22.48% to 36.65% for *P. commune* (PDLd10). The percentage of adhesion has increased using argan oil for all fungal strains (30.71% for *P. commune* (PDLd”), 22.42% for *T. hyalocarpa*, 28.39% for *P. Commune* (PDLd10), and 24.76% for *A. niger*) and confirmed the theoretical prediction of adhesion. In summary, the antiadhesive effect of vegetable oils is fungal strain dependent as well as oil dependent.

These results can be attributed to secondary compounds resulting from the oxidation of fatty acids that can be found on the wood surface as well as the vegetable oil (fatty acids) reactions with wood. In fact, vegetable oils with higher degree of saturation are more sensitive to oxidation reaction. Polyunsaturated fatty acids have an important degree of oxidation unlike monounsaturated fatty acids. These latter have a different oxidation process: the monounsaturated fatty acid reacts with wood elements and becomes immobilized unlike the polyunsaturated ones which oxidize without binding to wood [16].

The relationships between the XDLVO approach and the adhesion experiments realized by ESEM were not always positive in our study. A contradiction between theoretical predictions and the results of the adhesion tests was noticed. Several factors can explain this difference. According to the work in [50], the XDLVO theory does not take into account biological-specific interactions and takes into account only the Lifshitz–van der Waals and acid-base components responsible of first steps of adhesion. Other authors reported that the cause of these significant discrepancies is due to the non-DLVO interactions and physical and chemical heterogeneities [51–53]. So, microbial adhesion is a multifactorial phenomenon in which other factors could contribute other than the Lifshitz–van der Waals and acid-base interactions.

## 4. Conclusions

The treatment with different vegetable oils has increased the hydrophobicity quantitatively and the electron-donor component of the cedar wood surface. The antiadhesive effect of vegetable oils is fungal strain dependent as well as oil dependent. Among the tested oils, olive and linseed oils were seen to provide the best antiadhesive activity against *P. commune* (PDLd”) and *T. hyalocarpa*. Sunflower and rapeseed oils have also worked well against the accession of *P. commune* (PDLd10) and *A. niger*. In fact, the *P. commune* (PDLd”)/*T. hyalocarpa* adhesion after treatment with olive oil was governed by long-range forces (the van der Waals interactions) unlike the accession of *P. commune* (PDLd10)/*A. niger* which was governed by short-range forces (the acid-base interactions) after treatment with sunflower and rapeseed oils.

## Figures and Tables

**Table 1:** Chemical composition of different vegetable oils.

	Saturated fatty acid (g/100 g)	Monounsaturated fatty acid (g/100 g)	Polyunsaturated fatty acid(g/100 g)	Reference
Sunflower oil	10.4	28.2	57.5	*∗*
Rapeseed oil	6.98	60.5	26.3	*∗*
Linseed oil	9.4	20.2	66	*∗∗*
Olive oil	13.8	75.2	6.88	*∗*
Argan oil	17.6	44.8	33.3	*∗*

^*∗*^French food composition table Ciqual [23] ANSES, the French agency for food, environmental, and occupational health safety. ^*∗∗*^USDA Food Composition Databases [24], United States Department of Agriculture, Agricultural Research Service.

**Table 2:** Surface tension properties of pure liquids used to measure contact angles [28].

Liquid	*γ * ^*LW*^ (mJ/m^2^)	*γ * ^+^ (mJ/m^2^)	*γ * ^−^ (mJ/m^2^)
Water (H_2_O)	21.8	25.5	25.5
Formamide (CH_3_NO)	39	2.3	39.6
Diiodomethane (CH_2_I_2_)	50.5	0	0

**Table 3:** Contact angles values, surface energies, and their components of cedar wood before and after treatment.

	Contact angles (°)			Surface energy: components and parameters (mJ/m^2^)					Δ*G*_*iwi*_
	*θ*_*W*_(°)	*θ*_*F*_(°)	*θ*_*D*_(°)	*γ* ^*LW*^	*γ* ^+^	*γ* ^−^	*γ* ^*AB*^	*γ* ^Tot^	
Untreated wood	87.13 ± 0.15	66.61 ± 0.14	24.41 ± 0.47	46.44	0.67	4.47	3.36	49.8	−59.20
Treated with sunflower oil	64.95 ± 0.24	49.75 ± 0.38	14.35 ± 0.23	48.93	0.60	17.66	5.87	54.8	−25.84
Treated with rapeseed oil	73.95 ± 0.29	55.60 ± 0.09	18.25 ± 0.18	48.15	0.31	10.66	5.05	53.2	−43.45
Treated with linseed oil	66.35 ± 0.14	47.05 ± 0.43	13.45 ± 0.16	49.18	0.02	14.12	0.97	50.15	−37.10
Treated with olive oil	70.05 ± 0.29	53.20 ± 0.19	9.05 ± 0.86	49.99	0.26	13.58	4.01	54	−36.47
Treated with argan oil	68.25 ± 0.53	46.25 ± 0.20	14.40 ± 0.46	49.09	0.03	11.59	1.51	50.6	−43.44

**Table 4:** Contact angles values, surface energies, and their components for four fungal strains.

Strains	Contact angles (°)			Surface energy: components and parameters (mJ/m^2^)					Δ*G*_*iwi*_	Reference
	*θ*_*W*_(°)	*θ*_*F*_(°)	*θ*_*D*_(°)	*γ* ^*LW*^	*γ* ^+^	*γ* ^−^	*γ* ^*AB*^	*γ* ^Tot^		
*P. commune* (PDLd”)	36.11 ± 0.65	43.62 ± 0.75	51.31 ± 0.18	33.5	0.2	51.9	6.8	40.3	37.12	[31, 32]
*T. hyalocarpa*	41.90 ± 0.63	45.10 ± 0.19	55.00 ± 0.55	31.5	0.5	44.90	9.2	40.7	26.86	[32]
*P. commune* (PDLd10)	39.33 ± 1.13	31.73 ± 0.93	77.66 ± 0.42	18.66	7.97	36.06	33.9	52.56	8.28	[31, 32]
*A. niger*	48.31 ± 0.26	26.15 ± 0.21	15.05 ± 0.98	48.97	0.45	24.53	5.93	54.9	−12.57	This work

**Table 5:** The total interaction free energy ∆*G*^Tot^, the polar forces of Lewis ∆*G*^*AB*^, and apolar Lifshitz–van der Waals ∆*G*^*LW*^ of the adhesion of fungal strains studied for untreated and treated wood (in mJ/m^2^).

	*Penicillium commune* (PDLd”)			*Thielavia hyalocarpa*			*Penicillium commune* (PDLd10)			*Aspergillus niger*		
	Δ*G*^*LW*^	Δ*G*^*AB*^	Δ*G*^Tot^	Δ*G*^*LW*^	Δ*G*^*AB*^	Δ*G*^Tot^	Δ*G*^*LW*^	Δ*G*^*AB*^	Δ*G*^Tot^	Δ*G*^*LW*^	Δ*G*^*AB*^	Δ*G*^Tot^
Untreated wood	−4.80	−8.79	−13.60	−4.03	−11.53	−15.56	1.50	−4.99	−3.49	6.48	−26.54	−20.06
Treated with sunflower oil	−5.21	11.03	5.82	−4.37	7.07	2.70	1.62	4.58	6.20	7.02	−8.28	−1.26
Treated with rapeseed oil	−5.08	3.43	−1.65	−4.27	−0.28	−4.55	1.59	0.85	2.44	6.85	−16.54	−9.69
Treated with linseed oil	−5.25	9.37	4.12	−4.40	5.05	0.65	1.64	3.67	5.31	7.07	−12.39	−5.32
Treated with olive oil	−5.38	6.99	1.61	−4.51	3.12	−1.39	1.68	2.59	4.27	7.25	−12.87	−5.62
Treated with argan oil	−5.23	−41.8	−47.03	−4.39	−43.41	−47.80	1.63	−57.56	−55.93	7.05	−56.02	−48.97

## Data Availability

No data were used to support this study.
